# Chitosan Nanoparticles: A Versatile Platform for Biomedical Applications

**DOI:** 10.3390/ma15196521

**Published:** 2022-09-20

**Authors:** Showkeen Muzamil Bashir, Gulzar Ahmed Rather, Ana Patrício, Zulfiqar Haq, Amir Amin Sheikh, Mohd Zahoor ul Haq Shah, Hemant Singh, Azmat Alam Khan, Sofi Imtiyaz, Sheikh Bilal Ahmad, Showket Nabi, Rabia Rakhshan, Saqib Hassan, Pedro Fonte

**Affiliations:** 1 Molecular Biology Laboratory, Division of Veterinary Biochemistry, Faculty of Veterinary Sciences and Animal Husbandry, Shuhama Alusteng, Srinagar 190006, India; 2 Department of Biomedical Engineering, Sathyabama Institute of Science & Technology (Deemed to be University), Chennai 600119, India; 3 iBB—Institute for Bioengineering and Biosciences, Department of Bioengineering, Instituto Superior Técnico, Universidade de Lisboa, 1049-001 Lisboa, Portugal; 4 Associate Laboratory i4HB—Institute for Health and Bioeconomy, Instituto Superior Técnico, Universidade de Lisboa, Av. Rovisco Pais, 1049-001 Lisboa, Portugal; 5 ICAR-Poultry Seed Project, Division of LPM, Skuast-K 132001, India; 6 International Institute of Veterinary Education and Research (IIVER), Bahu Akbarpur, Rohtak 124001, India; 7 Laboratory of Endocrinology, Department of Bioscience, Barkatullah University, Bhopal 462026, India; 8 Department of Polymer and Process Engineering, Indian Institute of Technology, Roorkee 247667, India; 9 Large Animal Diagnostic Laboratory, Department of Clinical Veterinary Medicine, Ethics & Jurisprudence, Faculty of Veterinary Sciences and Animal Husbandry, Shuhama Alusteng, Srinagar 190006, India; 10 Department of Microbiology, School of Life Sciences, Pondicherry University, Puducherry 605014, India; 11 Center for Marine Sciences (CCMAR), Gambelas Campus, University of Algarve, 8005-139 Faro, Portugal; 12 Department of Chemistry and Pharmacy, Faculty of Sciences and Technology, Gambelas Campus, University of Algarve, 8005-139 Faro, Portugal

**Keywords:** drug delivery, nanocarrier, target therapy, chemotherapy, gene therapy, tissue engineering

## Abstract

Chitosan is a biodegradable and biocompatible natural polymer that has been extensively explored in recent decades. The Food and Drug Administration has approved chitosan for wound treatment and nutritional use. Furthermore, chitosan has paved the way for advancements in different biomedical applications including as a nanocarrier and tissue-engineering scaffold. Its antibacterial, antioxidant, and haemostatic properties make it an excellent option for wound dressings. Because of its hydrophilic nature, chitosan is an ideal starting material for biocompatible and biodegradable hydrogels. To suit specific application demands, chitosan can be combined with fillers, such as hydroxyapatite, to modify the mechanical characteristics of pH-sensitive hydrogels. Furthermore, the cationic characteristics of chitosan have made it a popular choice for gene delivery and cancer therapy. Thus, the use of chitosan nanoparticles in developing novel drug delivery systems has received special attention. This review aims to provide an overview of chitosan-based nanoparticles, focusing on their versatile properties and different applications in biomedical sciences and engineering.

## 1. Introduction

Nanoparticles are made of different materials, with a size range of 1–1000 nm considered for nanomedicine and biomedical applications. Nanoparticles can be produced by top-down procedures, such as sonication, high-pressure, and homogenization, or bottom-up processes such as solvent displacement and reactive precipitation [1]. They have unique properties, being smaller at the atomic level than their bulk counterparts [2]. As a result, nanoparticles with desired properties can be produced [3]. The two types of nanoparticles are organic and inorganic, with inorganic nanoparticles gaining in popularity because of their ability to survive harsh processing conditions [4]. Due to their changeable optical properties and physical endurance, metal oxide nanoparticles, including zinc oxide, magnesium oxide, silver oxide, and titanium oxide, have attracted significant attention among inorganic materials [5]. In addition, due to their unique electrical, metallurgical, and structural properties, organic materials, such as lipids, polymers, and carbon nanotubes, have a wide range of applications [6]. Because of their stability and ease of surface modification, natural and synthetic polymers can be used to create polymeric nanoparticles. Biopolymeric nanoparticles provide additional advantages such as biodegradability, biocompatibility, and reduced toxicity. Moreover, these nanoparticles are readily available from natural sources (e.g., cellulose, pectin, starch, collagen, silk fibroins, chitosan, and chitin).

Chitosan, a biodegradable and biocompatible polymer derived from the deacetylation of chitin, has a cellulose-like carbohydrate foundation structure with two types of alternating repeating units, glucosamine units and N-acetyl glucosamine, linked by a 1-4-glycosidic linkage, and is a whitish and inelastic polysaccharide [7]. It is used in farming, the food industry, biomedical applications, water treatment, pollution control, photography, paper making, and many other uses [8]. Chitosan nanoparticles (ChNP) include features and advantages of both chitosan and nanoparticles [9]. Because it is inexpensive and widely accessible, chitosan has a broad range of uses, particularly in medicine, such as wound healing [10] and in the production of drug delivery systems [11]. Chitosan also has mucoadhesive characteristics, which enables ChNP to be delivered by transmucosal routes including intranasal, intraocular, intravaginal, intratracheal, intrapulmonary, and others [12]. Chitosan is also used in agriculture to produce fertilisers [13] and is commonly present in food coatings [14]. It is also advertised as a dietary fibre in some countries, such as the USA, to reduce fat absorption [15] and it finds use in cosmetic products as a skin moisturiser [16]. Chitosan products, made by altering their essential structure for producing polymers with various properties [17], are crucial for application diversification. For example, polymeric nanoparticles containing chitosan have been employed to deliver drugs by different delivery routes. The positive surface charge and mucoadhesive properties of nanoparticles made of chitosan and chitosan derivatives enable them to attach to mucous membranes and release the loaded drug over time [18]. ChNP have several uses in non-parenteral drug administration for the treatment of eye infections, cancer, gastrointestinal illnesses, respiratory diseases, cancer, and others [19]. Based on these considerations, we believe that chitosan is a naturally occurring material with the greatest potential for use in nanomedicine.

Significantly, chitosan is a material that can be processed in multiple ways to produce a variety of three-dimensional scaffolds with different pore structures for use in bone tissue engineering. It can also be combined with a variety of materials, including ceramics and polymers, to yield composite scaffolds with superior mechanical and biological properties. The suitability of chitosan nanoparticles for peptide or growth factor delivery in bone tissue engineering has also been described [18]. Chitosan nanoparticles can be successfully synthesised using a modified precipitation process with NaOH as a precipitant.

The aim of this review is to provide an overview of the usefulness of ChNP for biomedical applications such as drug delivery, vaccine development, tissue engineering, and others. Their production methods, as well as their performance as delivery systems, are fully disclosed.

## 2. Properties of Chitosan for Biomedical Applications

Chitin is the second most common natural polysaccharide globally behind cellulose [16]. Chitosan quality is determined by the chitin source, its separation, and the degree of deacetylation [19]. Many aquatic and terrestrial species, as well as certain microbes, are the main sources of chitin and chitosan [20]. The processing of marine organisms (e.g., shrimp, lobster, crab, and squid) generates biowaste, which is used to obtain chitosan and chitin [21,22]. However, due to seasonal and variable raw material supplies, unpredictability and issues in processing conditions have hampered the industrial production of chitosan from biological waste generated by aquatic organisms [22,23]. As a result, terrestrial species, such as terrestrial crustaceans, mushrooms, and insects, have been used to obtain chitosan as a partially deacetylated polymer [24]. Silkworms and honeybees have been given special attention among terrestrial creatures since the waste products from creatures looked to be a promising source for chitosan and chitin production on a massive scale [25]. Yeasts, moulds, certain chrysophyte algae, ciliates, and several bacteria, notably *streptomycetes* pores and *Prosthecate bacterium* stalks, contain both chitin and chitosan in the microbial organisms [26]. The ability to synthesise chitosan from microorganisms seems to be a good option, as the technology can be altered to generate a clean and homogeneous product with specified qualities [27]. Despite the fact that chitosan and chitin can be produced from various terrestrial/aquatic micro- and macroorganisms, their economic use is limited to a few species [28].

**Figure 1:**
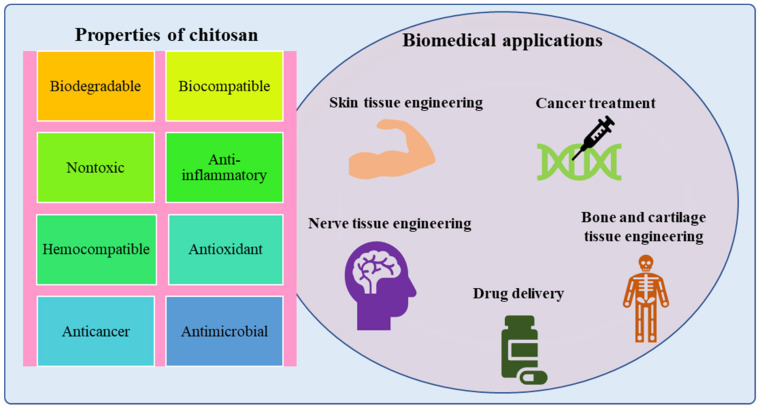
Properties of chitosan-based nanoparticles and their biomedical applications.

The different properties of chitosan make it an excellent biomaterial for various biomedical applications as shown in Figure 1. One of the most notable properties of chitosan is that it does not cause severe inflammation or stimulation of the immune system. Chitosan with various deacetylation degrees and molecular weights has low toxicity [29,30]. Chitosan has bactericidal properties because of the cationic nature of the polymer since microbial growth is prevented by the adhesion of the positively charged polymer to the bacterial surfaces that induce changes in the permeability of the membrane wall [31]. Chitosan with lower deacetylation and a low pH has improved antibacterial activity. The antibacterial action against Gram-negative bacteria can be amplified and decreased against Gram-positive bacteria by reducing the molecular weight [32]. The hydrophilic nature of the cell wall is essential for chitosan to interact with the bacterial cell, which may explain why chitosan is minimally harmful to mammalian cells [29]. Another important feature is chitosan’s mucoadhesive properties, which leads to new ways of delivering drugs through mucosal routes and aids in the adsorption of compounds with no affinity for mucus [33]. Chitosan increases drug permeation through epithelia by helping in the opening of the tight epithelial junctions [31]. Chitosan has been also widely used in coagulation studies since it interacts with platelets and amino groups on the chitosan surface to enhance wound healing [34]. Chitosan’s haemostatic properties have long been used in wound healing. Chitosan can activate macrophages and neutrophils, accelerate granulation tissue, cause re-epithelisation, lessen scar formation and contraction, and cause haemostasis and also has features such as chemoattraction, analgesic properties, and intrinsic antimicrobial qualities as a wound dressing material [35].

Chitosan is a semi-crystalline polysaccharide composed of linearly arranged N-acetyl-d-glucosamine and d-glucosamine residues. Because of the presence of an amino group (-NH_2_) in its structure, it is cationic in nature. This positive charge promotes extracellular matrix formation by attracting negatively charged molecules such as proteoglycans [36]. Furthermore, a hydroxyl group (-OH) is present in the structure and captivates positively charged molecules to improve bonding [37]. Apart from electrostatic attraction, these functional groups aid in the modification of chitosan, enhancing its mechanical and physical properties and resulting in novel functional characteristics and convincing clinical relevance [38].

Chitosan is formed via the precipitation of polyanions in alkaline solutions. Although it has medical properties, such as anti-ulcer [39], wound healing [40], and antibacterial properties, as well as the ability to reduce cholesterol levels [41], further research is needed [42,43]. The cationic nature of the R-NH_3_^+^ group of chitosan confers mucoadhesive qualities while interacting with negatively charged groups of mucosal surfaces [44]. When protonated amine groups interact with the cell membrane, protein-associated tight junctions undergo reversible structural remodelling, followed by tight junction opening. Another property that distinguishes chitosan from other polysaccharide polymers is the ease with which the structure may be chemically modified, particularly in the C-2 position, resulting in derivatives with distinct properties and possible uses [45,46].

Recent research has also found that chitosan and its derivatives depict anticancer properties both in vitro and in vivo. Chitosan derivatives may have anticancer properties because they stimulate cytolytic T-lymphocyte maturation and infiltration by increasing the concentration of interleukin (IL)-1 and -2 secretions [47]. Antioxidants are well-known for their beneficial effects on human health. They protect membrane proteins, lipids, and DNA against the free radicals of the body [48]. Chitosan and its derivatives have shown free radical scavenging capability in vitro. Low-weight chitosan has various benefits over high-molecular-weight chitosan when it comes to eliminating free radicals [49]. According to Zhao et al., amino and carboxyl groups stabilising free radicals may provide chitosan with an antioxidant effect [50].

Other especially important properties are chitosan’s biodegradability and biocompatibility since in biological organisms, bioenzymes can catalyse the depolymerisation of chitosan. Humans are unaffected by the breakdown products N-acetyl glucose and glucosamine, and the degradation intermediates may not remain and are not allergenic to the body.

## 3. Production of Chitosan Nanoparticles

ChNP are made from chitosan or its derivatives. The N-deacetylated derivative of chitin is an appealing biopolymer for producing nanoparticles because chitosan has a unique polymeric cationic nature, non-toxicity, high biocompatibility, mucoadhesive properties, absorption-enhancing qualities, and biodegradability [51]. Chitosan’s cationic nature allows ionic cross-linking with multivalent anions [52] and its linear polyamine structure has various free amine groups that are obtainable for cross-linking, which are important factors that make it useful in the production of nanoparticles. ChNP have unique characteristics that allow for greater affinity for negatively charged biological membranes as well as in vivo site-specific targeting [53]. As a result, they can be used to effectively load drugs, enzymes, and nucleic acids [54,55] using a controlled release [56] for several applications in different industries. ChNP have exceptional chemical, morphological, and physical properties determined by the material features and the production technique. Chitosan is insoluble in water and soluble in solutions containing acids such as citric, tartaric, and acetic acids [57,58,59]. It is available in low- and high-molecular weights ranging from 3800 to 20,000 Da. The degree of deacetylation and molecular weights of chitosan significantly affect its features, most notably during the formation of nanoparticles. Anticancer drugs, antimicrobials, peptides, anti-inflammatories, growth factors, and other pharmaceuticals have been successfully delivered using chitosan-based polymeric drug carriers [60]. 

ChNP boost the capacity of bioactive compounds to dissolve, entrap, encapsulate, and/or cling to the nanoparticle matrix. These systems have large surface areas where bioactives can be adsorbed. Their nanoscale size also improves efficient penetration through epitheliums. ChNP can also carry drugs, proteins, and DNA with low-to-high molecular weights and are negatively charged for targeting organs, cells, and tissues [61]. ChNP are also suitable for mucosal distribution, such as nasal, oral, and ocular mucosa, due to their characteristics and functions. When chitosan encounters anions, it forms a gel and beads and this feature allows it to be used in drug delivery. In addition, the size of the beads (1–2 mm) restricts its applicability [62,63]. Ohya and colleagues described ChNP for the first time in 1994 and employed emulsified and cross-linked ChNP to deliver the antitumor drug 5-fluorouracil intravenously [64,65]. To date, different techniques have been developed to produce ChNP and some of them are briefly discussed herein [66]. Overall, the most common techniques are ionotropic gelation and polyelectrolyte complexation since they are straightforward and do not need large shear forces or organic solvents [67].

### 3.1. Ionotropic Gelation

**Figure 2:**
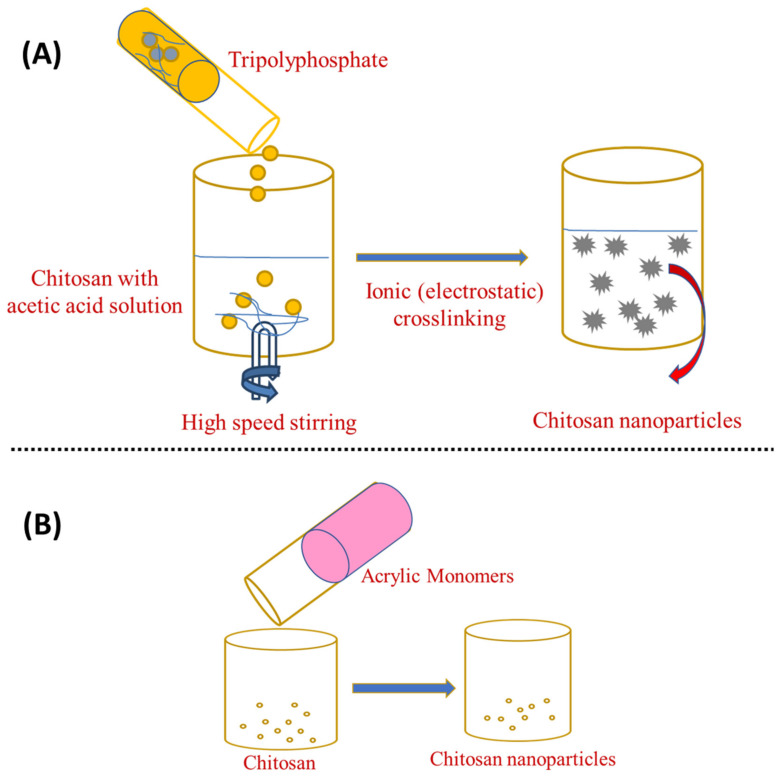
Preparation of chitosan nanoparticles by (**A**) ionotropic gelation and (**B**) ionotropic gelation with the radical polymerisation method.

Chitosan can be cross-linked physically and chemically to generate nanoparticles because its backbone contains a number of amine groups that are protonated to form NH_3_^+^ in acidic conditions [68]. Physical cross-linking has sparked a lot of attention over chemical cross-linking as it eliminates toxic substances, reduces undesirable effects, and improves biocompatibility [52,69]. In addition, this simple and mild procedure allows for real cross-linking [70]. Physical cross-linking depends on the chitosan (positive charge) and multivalent ions (negative charge) generated by sodium tripolyphosphate (TPP) [71], citrate, and sulphate [72]. Calvo et al. were the first to report this ionic gelation process, which has been extensively studied and refined [73]. When chitosan gelation takes place by tiny anionic molecules, such as phosphate, citrate, or sulphate, the designated ionic gelation is used. When anionic macromolecules are used instead of tiny molecules, a polyelectrolyte complexion is thought to occur [74]. This approach makes use of the electrostatic interaction among the amine group of chitosan and a negatively charged polyanion such as tripolyphosphate [75]. Chitosan is poured into an acetic acid solution or added with a stabilising agent, such as poloxamer, and the tripolyphosphate aqueous solution is mixed under vigorous stirring. Then, anionic particles diffuse into the chitosan molecules and cross-linking occurs, leading to nanoparticle formation with a size range of 200–1000 nm, as shown in Figure 2A. After a couple of centrifugations and washing with water, ChNP are collected by freeze-drying or oven-drying. In this process, the chitosan-to-stabiliser ratio can alter the nanoparticles’ surface charge and size [76,77,78]. Increasing the chitosan-to-polyanion ratio results in an increase in particle size [79]. Since the smaller particle size was revealed in sodium chloride, nanoparticles disseminated in saline solution were also found to be more stable. The electrostatic repulsion between the amine groups of the chitosan backbone is reduced when a monovalent salt (sodium chloride) is added to the solvent. The polymer chains become more flexible, enhancing their stability [80].

Ionotropic gelation can also be combined with radical polymerisation, which causes chitosan to gel, whereas acrylic or methacrylic acid is polymerised [81]. As shown in Figure 2B, potassium persulfate is used as an initiator for the polymerisation reaction, which takes around 6 h to complete at 60–70 °C [75]. This approach eliminates the unreacted particles by dialysis or washing with water. Silk peptide, insulin, and serum albumin have been successfully loaded using this approach [82]. Overall, the ionotropic gelation method is the simplest and most cost-effective from bench-to-industrial scale-up. This is because this method requires simple, inexpensive materials and equipment and it can be easily accomplished moderately and rapidly in conventional research labs. Furthermore, the framework premised on electrostatic interaction rather than chemical reaction eradicates the need for organic solvents, avoiding unnecessary toxicological effects. Encapsulation efficiency can also be improved when the technique is set up to achieve optimised polymer–drug interactions [67].

### 3.2. Emulsion Droplet Coalescence and Emulsion Solvent Diffusion

**Figure 3:**
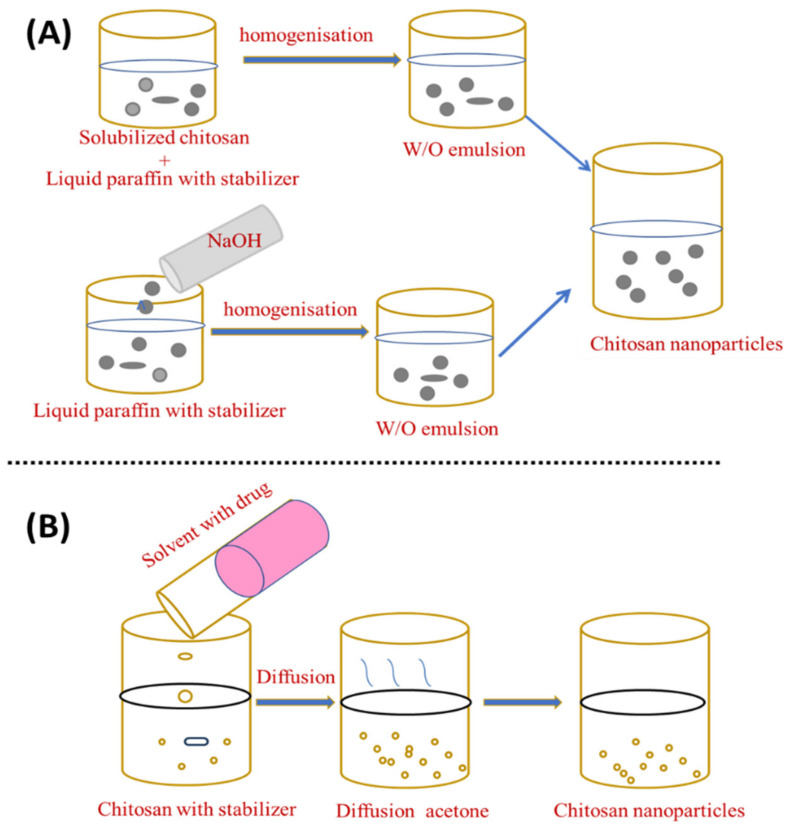
Preparation of chitosan nanoparticles by (**A**) emulsion droplet coalescence and (**B**) emulsion solvent diffusion.

In the emulsion droplet coalescence, a stable water-in-oil emulsion is made by homogenising an aqueous chitosan solution with the drug in liquid paraffin including a stabiliser, such as Span^TM^ 83, at high speed. During mixing both emulsions, droplets from both emulsions will collide and coalesce, causing chitosan droplets to precipitate and form nanoparticles of about 452 nm, as shown in Figure 3A [83].

El-Shabouri (2002) was the first to report on the emulsion solvent diffusion method [84] using a modified approach established by Niwa et al. [85]. Initially, an oil-in-water type emulsion is created by injecting an organic phase, such as acetone or methylene chloride, into a solution containing the hydrophilic drug to an aqueous chitosan solution containing a stabilising agent, such as lecithin/poloxamer, with stirring followed by high-pressure homogenisation to evaporate methylene chloride. By diffusing acetone into the aqueous phase, the solubility of chitosan is decreased and the polymer precipitates, owing to the organic solvent diffusion into the water, resulting in nanoparticles with an average size of between 100 and 500 nm [86], as shown in Figure 3B. An excess amount of water is added to ensure complete acetone diffusion and the nanoparticles are separated using centrifugation. This novel approach can reduce the particle size and distribution of the synthesised ChNP. Although the emulsification strategy leads to better particle size control, strong cross-linking agents are generally used in this procedure and the total elimination of the residual cross-linking agents can be challenging [15]. The emulsion solvent diffusion method is appropriate for both hydrophobic and hydrophilic drugs. In the case of hydrophilic drugs, a multiple water/oil/water emulsion (e.g., double emulsion) with the drug dissolved in the internal aqueous phase should be established. However, the use of high shear forces and organic solvents required during nanoparticle formation are two drawbacks of this approach.

### 3.3. Reverse Micellar Method 

The reverse micellar approach is used to produce polymeric nanoparticles with a narrow size distribution [85]. Different polymers can be used to obtain micelles to carry drugs. Reverse micelles are thermodynamically stable liquid mixtures of water, oil, and surfactant. Compared to the typical emulsion polymerisation processes, the reverse micelle-hosted technology has a dynamic behaviour due to the generation of particles with a narrow size range [52]. This method uses a surfactant dissolved in an organic solvent to create reverse micelles. The Brownian motion randomly shifts the micellar droplets so they divide into two micelles after swapping their water content [87].

**Figure 4:**
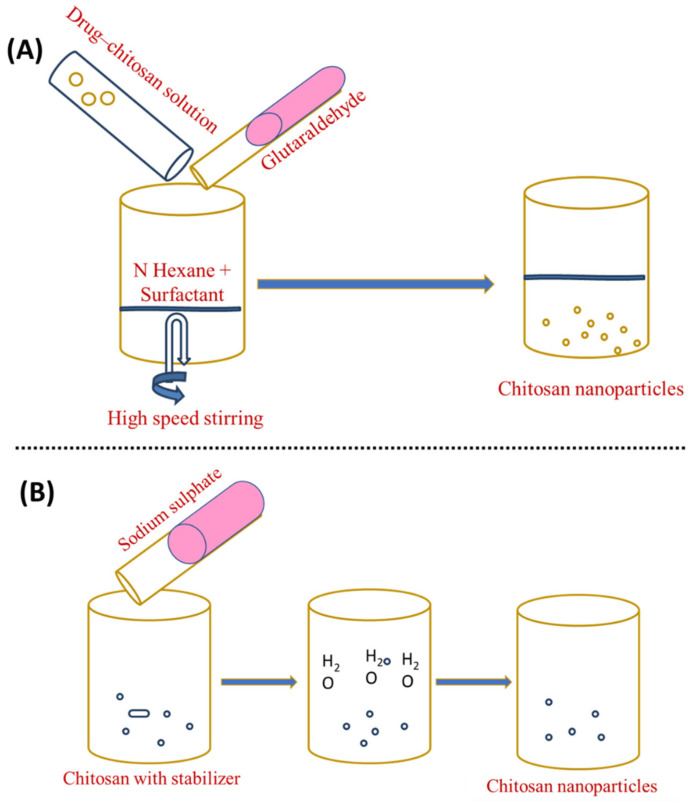
Preparation of chitosan nanoparticles by (**A**) reverse micellisation and (**B**) desolvation.

To prepare an organic phase, a lipophilic surfactant, such as sodium bis-(2-ethylhexyl) sulphosuccinate, is mixed in an organic solvent such as n-hexane (water in oil emulsion). After that, an aqueous drug–chitosan solution is added to the surfactant and organic solvent while stirring. After adding a cross-linking agent, the liquid is stirred overnight for cross-linking. Then, the organic solvent is eliminated, leaving a dry substance. The dried material is added to water to eliminate the surfactant and salt is applied to remove the surfactant, followed by centrifugation to recover the ChNP. The isolation of the nanoparticles is accomplished in three steps: surfactant precipitation with CaCl_2_, dialysis to remove unreacted components, and freeze-drying [88]. This allows the production of small, narrow particle sizes. The steps in this strategy are depicted in Figure 4A. Bovine serum albumin (BSA)-loaded ChNP in the 80–180 nm size range were prepared using the reverse micellar technique [89].

### 3.4. Desolvation

The desolvation process was employed for the first time to manufacture micron-sized carriers [66]. Chitosan nanoparticles are frequently made using a process modified from sodium sulphate [90]. DNA and protein distribution is possible using this method [91]. Phase separation and coacervation are the foundations of the desolvation technique. Nanoparticles precipitate when desolvation agents (such as ethanol or acetone) are added, and nanoparticle stability is achieved by adding a cross-linking agent [92]. In this process, an aqueous chitosan solution with a stabilising agent (e.g., Tween 20) is treated with a precipitation agent (e.g., sodium sulphate). The salty chitosan solution causes a steady removal of water-encircling chitosan. Due to the insolubilisation of chitosan, precipitation occurs. To harden the nanoparticles with an average range of 373 ± 71 nm, glutaraldehyde is applied last [52,93,94]. This technique has several advantages over other methods, the most notable of which is its ability to create nanoparticles in a single step, in addition to its low costs, low use of electricity, and frequency [95,96]. Figure 4B shows an illustration of this strategy.

### 3.5. Nano Precipitation

**Figure 5:**
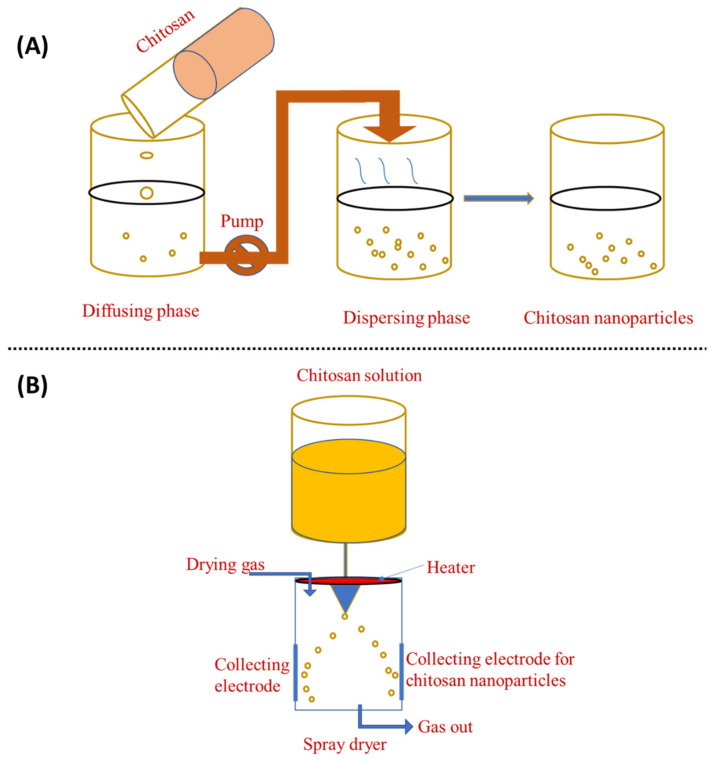
Preparation of chitosan nanoparticles by (**A**) nanoprecipitation method and (**B**) spray-drying method.

The solvent displacement technique, commonly referred to as nanoprecipitation, has many benefits over other approaches. The principle of this fabrication method is known as the Marangoni effect. In the nanoprecipitation method, the nanoparticles are obtained from the colloidal suspension when the oil phase is slowly added to the aqueous phase under moderate stirring. The formation of the nanoparticles is instantaneous and needs only one step so it has the advantage of a rapid and straightforward operation. The key parameters in the fabrication procedure have a great influence on the nanoprecipitation method such as the organic phase injection rate, aqueous phase agitation rate, and the oil phase/aqueous phase ratio [97]. Particle sizes with very narrow distribution can be synthesised because of the absence of shearing stress. This method is used mostly for hydrophobic drug entrapment but is sometimes employed to incorporate hydrophilic drugs. Polymers and drugs are dissolved in a water-miscible organic solvent, for example, acetone or methanol. The solution is then added to an aqueous solution that contains a surfactant in a drop-wise manner. Through rapid solvent diffusion, the nanoparticles are formed immediately. Afterwards, the solvents are removed under reduced pressure. A diffusing phase is created by dissolving chitosan in a solvent system and injecting it into the dispersion phase, i.e., methanol, through the membrane using a peristaltic pump at a constant flow rate of 0.8 mL/min. Tween 80 is mixed into the dispersion phase to obtain nanoparticles [66]. The nanoprecipitation approach can also generate nanoparticles with sizes ranging from 50 to 300 nm, which is advantageous because smaller particle sizes generate more areas of contact. This property is critical for its use in adsorption and desorption systems [98,99]. This method can generate particles as small as 170 nm, which increases the number of applications as well as its efficiency. Figure 5A shows a schematic representation of this method.

### 3.6. Spray-Drying

Spray-drying is another method for producing ChNP, as shown in Figure 5B. In this process, a nano spray dryer is used. Chitosan is solubilised with glacial acetic acid in water, which is then stored overnight. The solution is then atomised, which helps to generate droplets using an atomiser. The liquid phase is then evaporated by mixing these droplets with a drying gas, resulting in the formation of ChNP. Generally, the spray-drying nozzle size is 4.0, 5.5, or 7.0 μm and the flow rate is 2 mL/min; the drying gas flow is 1.3 m^3^/min, the inlet temperature 120 °C, and the outlet temperature 80 °C [100]. In the spray-drying process, the features and manufacturing yield of ChNP are influenced by the original feed, as well as the operating parameters, such as flow rate, nozzle size, and inlet and outlet temperatures [81]. In the pharmaceutical industry, spray-drying is frequently used to create the microencapsulation of antibiotics such as ampicillin, amoxicillin, vancomycin, etc [81]. Spray-drying is a simple, one-stage, continuous process that is only sluggishly influenced by the solubility of the drug and polymer. It can also be employed with pharmaceuticals that are heat-resistant, heat-sensitive, water-soluble, or water-insoluble and for hydrophilic or hydrophobic polymers [100]. Spray-drying is a simple, one-step approach that is protein-friendly for protein-loaded ChNP [101]. Ozturk et al. [102] used spray-drying to create ChNP comprising dexketoprofen and evaluated them for anti-inflammatory activity. ChNP loaded with dexketoprofen trometamol appear to be a potential oral prolonged-release medication delivery strategy with low dosages and good efficiency.

## 4. Characterisation of Chitosan Nanoparticles

The ChNP must be evaluated in terms of their loading efficiency, in vitro release, particle size and zeta potential analysis, morphological and surface characteristics, and characterisation of the desired therapeutic outcome. Particle size is an essential physical feature to evaluate so the ability to analyse and characterise it with devices in the sub-nanometre-to-millimetre range has proven to be crucial for the success of the production procedure.

The morphological and surface characteristics of nanoparticulate systems are typically assessed by a scanning electron microscope (SEM), which involves scanning the sample with a focused electron stream. Then, it produces various signals detected by a sensitive detector, which offers information about the sample surface as an image whose resolution has no diffraction limit. Before being examined by SEM, dried particle samples are mounted in a metal stub and coated with gold or another conductive material under vacuum-drying. SEM can also reveal surface roughness/smoothness or porous structures, which can aid in the interpretation of future analyses such as dissolution behaviour or in vivo reactions [103]. The morphology and occurrence of chitosan aggregates [104] and alginate/ChNP have been studied using SEM [105]. A transmission electron microscope (TEM) is also used for the analysis of chitosan-based nanoparticles. It employs a high-energy beam of accelerated electrons that passes through a nanomaterial sample [106]. Sample preparation in a TEM entails suspending chitosan-based nanomaterials on a carbon film-covered copper grid, accompanied by drying before observation [106]. TEM can resolve down to 0.2 nm under ideal conditions [50,107].

Recently, cryogenic-scanning electron microscopy coupled with transmission electron microscopy (cryo-SEM/TEM) has become a valuable tool for the characterisation of nanomaterials. At ultra-low temperatures, this technique enables the analysis of nanomaterials that have not been chemically modified and are fully hydrated. It can be used to examine the surface appearance, shape, size, and internal structure of chitosan-based nanomaterials in the same way as SEM and TEM. Because of the rapid rate of the sample preparation, speed, convenience of use, and image-processing effectiveness, this technique is being used more widely [108]. The above method removes the necessity of conventional sample preparation, such as critical point drying. Cryo-SEM/TEM has several advantages over traditional SEM or TEM, including its high-resolution capability, rapidity, analysis in the fully hydrated state, less relocation of diffused materials, and suitability for liquid or semi-liquid materials.

The degree of electrostatic repulsion between charged particles is reflected in the magnitude of the zeta potential. In a dispersion system, a high zeta potential, whether negative or positive, implies that the particles are resistant to aggregation and the colloidal system appears to be stable [109]. Rodrigues et al. [110] used laser Doppler anemometry to assess the zeta potential of chitosan and carrageenan nanoparticles. In nanoparticle production, there are parameters, such as the loading and encapsulation efficiency, nanoparticle yield, and drug content, which should be estimated before therapeutical administration. An appropriate drug analysis technology, such as spectrophotometry or high-performance liquid chromatography (HPLC), should be used to determine the drug content. The following equations are used to determine the drug encapsulation efficiency, loading efficiency, and nanoparticle yield.

$$\mathrm{Encapsulation}\;\mathrm{efficiency}\;\left(\%\right)=\frac{\mathrm{Drug}\;\mathrm{loaded}\;\mathrm{into}\;\mathrm{nanoparticles}\;\left(\mathrm{mg}\right)}{\mathrm{Theoretical}\;\mathrm{amount}\;\mathrm{of}\;\mathrm{drug}\;\left(\mathrm{mg}\right)}\times 100$$

$$\mathrm{Loading}\;\mathrm{efficiency}\;\left(\%\right)=\frac{\mathrm{Drug}\;\mathrm{loaded}\;\mathrm{into}\;\mathrm{nanoparticles}\;\left(\mathrm{mg}\right)}{\mathrm{Total}\;\mathrm{mass}\;\mathrm{of}\;\mathrm{nanoparticles}\;\left(\mathrm{mg}\right)}\times 100$$

$$\mathrm{Yield}\;\left(\%\right)=\frac{\mathrm{Total}\;\mathrm{weight}\;\mathrm{of}\;\mathrm{obtained}\;\mathrm{nanoparticles}\;\left(\mathrm{mg}\right)}{\mathrm{Total}\;\mathrm{weight}\;\mathrm{of}\;\mathrm{drug}\;\mathrm{and}\;\mathrm{polymer}\;\mathrm{used}\;\left(\mathrm{mg}\right)}\times 100$$

The drug is retrieved from nanoparticles and the amount of the released drug is measured to instantly ascertain the drug content by centrifuging the nanoparticle suspension at a high speed (up to 100,000 rpm, depending on nanoparticle size), collecting the supernatant with the unloaded drug, and then assessing the amount of the drug in the supernatant.

The particulate components, surface texture, complex formation, and other molecular information of the nanoparticles are investigated using X-ray photoelectron spectroscopy (XPS) and time-of-flight secondary ion mass spectrometry (ToF-SIMS). XPS identifies the patterns and chemical states according to the size of the particles. ToF-SIMS gives a detailed analysis of the surface and near-surface composition, illustrating the particles’ surface chemistry characterisation. Casettari et al. [111] used XPS and ToF-SIMS to investigate the presence of chitosan on the surface of manufactured PLGA/chitosan particles. In another study, XPS and ToF-SIMS were used to analyse the chemical composition and surface morphology of chitosan and carrageenan nanoparticles [110].

The drug-release behaviour of drug-loaded ChNP is determined using an in vitro release test, which can be influenced by the drug on the particle surface, drug diffusion from the chitosan matrix, and chitosan breakdown and erosion. Depending on the particle properties, the degree of cross-linking, presence of enzymes, excipients in the formulation, pH, and polarity, the drug release is generally diffusion-regulated and follows the Higuchi model [52,112]. The drug-release mechanism comprises water penetrating the polymeric particle system, causing the matrix to expand, followed by the creation of a rubbery polymeric matrix and the diffusion of the drug from the inflated flexible polymeric matrix. Large-sized particles, generated with a high chitosan concentration in each pH medium, could be used to deviate the drug release from diffusion to zero order. In a pH 7.4 release medium, indomethacin-loaded chitosan carriers displayed this behaviour [113].

## 5. Chitosan Nanoparticles in Biomedical Applications

Polymeric nanoparticles are widely used in the field of biomedicine as tools for disease diagnosis and treatment [114]. They can be loaded with different drugs and act as a delivery carrier, allowing for more effective drug delivery. Drugs can also be attached to the surfaces of polymeric nanoparticles. The capacity of these polymeric nanoparticles to target molecules with particular cell surface receptors and penetrate cells opens up new possibilities for drug delivery and gene therapy [113]. Because of their small nonspecific protein adsorption properties, polymeric nanoparticles with hydrophilic surfaces are exploited as carriers. They can also be used to diagnose complex health problems. Chitosan has numerous applications because of its unique characteristics, but it is insoluble in aqueous solutions, which is a significant drawback that restricts its use in biological systems [115].

Still, chitosan has functional groups that help graft molecules, giving the modified chitosan unique features. Chemical modifications can be used to improve chitosan solubility and, as a result, expand its applicability. Many types of chitosan derivatives are produced due to these chemical changes, and they are nontoxic, biocompatible, and biodegradable [116]. Additionally, ChNP can boost the immune system’s ability to treat cancer [117]. Therefore, ChNP are employed as drug carriers due to their high biocompatibility, biodegradability, and ease of modification [118]. 

ChNP have a diverse range of uses such as in drug and vaccine delivery, as vaccine adjuvants, as antibacterials, in tissue engineering, etc. This section describes some applications of ChNP in the biomedical field, which are briefly summarised in Table 1.

### 5.1. Drug Delivery

Chitosan and chitosan derivative-based nanoparticles have mucoadhesive properties and positive surface charges, allowing them to stick to mucus surfaces and provide sustained drug release. This is important for non-invasive drug delivery through pulmonary, nasal, oral, or vaginal routes [131]. Simultaneously, nanoparticles have emerged as a feasible drug delivery strategy, allowing for controlled release, drug protection from environmental or enzymatic degradation, and localised retention. Because of their low toxicity, mucoadhesion, and customisable physical characteristics, ChNP are particularly suited for oral, nasal, and pulmonary delivery. Both hydrophilic and hydrophobic drugs can be delivered using ChNP [132].

Oral delivery is undoubtedly the most comfortable method of delivery due to its simplicity and convenience. However, oral administration is hampered by harsh pH conditions, the availability of enzymes, first-pass metabolism on the liver, as well as intestinal resistance to drug absorption. In fact, all of these natural barriers decrease oral bioavailability because they prevent the drug from reaching the bloodstream [133]. Thus, nanoparticles are popular carriers for circumventing the limitations of oral drug delivery. In addition to these benefits, nanoparticles can improve the stability of acid-labile drugs in the gastrointestinal tract compared to alternative drug delivery methods such as lipid-based and liposome systems [54]. 

Green tea contains flavonoids such as catechin and epigallocatechin, which are powerful antioxidants. These are degraded in the presence of intestinal fluids and absorbed inadequately across the intestinal membranes so encapsulating catechin and epigallocatechin gallate in ChNP improves their intestinal absorption [134,135]. Tamoxifen, an anti-cancer medicine, is a suitable option for oral drug delivery because it is slightly water-soluble. Tamoxifen permeation across the intestinal epithelium was improved by combining it with lecithin and ChNP [136,137]. The nanoparticles are mucoadhesive, allowing more tamoxifen to pass through the paracellular route. Feng et al. also reported on an anti-cancer drug delivery approach that could be used orally [138]. They used chitosan and carboxymethyl chitosan to encapsulate doxorubicin hydrochloride (DOX). The intestinal absorption of DOX was reported to be enhanced by this delivery system. Alendronate sodium is an osteoporosis drug that has adverse gastrointestinal effects that lead to low oral bioavailability. The formulation of ChNP using an ion gelation technique resulted in a high nanoparticle encapsulation effectiveness of alendronate sodium of 13.24%. They determined that 80% of the drug was released in a 0.1 M HCl solution within 60 min and only 40% was released in a PBS solution (pH 6.8) within 4 h. The conclusion was that the drug release is pH-dependent [139]. This emphasises the need to assess the degree of the surface coverage of nanoparticles with chitosan during formulation development and conduct experiments in biorelevant environments. In another study, ChNP were produced using an ion cross-linking technique to deliver sunitinib, a tyrosine kinase inhibitor, efficiently and the system presented a 98% encapsulation efficiency and a sustained drug release was achieved for up to 72 h [140]. In one of the trials, tripolyphosphate was used to cross-link chitosan that had been loaded with insulin. The cross-linking process reduced the particle size and freeze-drying increased the stability of the nanoparticles. To ensure the stability of nanoparticles, freezing stresses throughout freeze-drying should be managed [140]. The gut epithelium showed considerable nanoparticle uptake but the system was unstable at gastric pH, indicating that more work is needed to develop a stable oral insulin delivery system [141]. Therefore, drug formulation using ChNP is becoming increasingly common for boosting drug solubility and oral bioavailability due to its low cytotoxicity and higher drug uptake than nanoparticles. For instance, Bay 41-4109, an active inhibitor of the hepatitis B virus, was incorporated into ChNP to improve its oral bioavailability [142].

The antigen breakdown in the gastrointestinal tract makes it difficult for vaccines to reach Peyer’s patches, the lymphoid tissue in the intestine. ChNP-based vaccines must be produced without using organic solvents that could change the immunogenicity of antigens [138]. Chitosan and carboxymethyl-ChNP have been demonstrated to be effective carriers for administering extracellular *V. anguillarum* products in oral vaccines. The nanoparticles were stable at gastric pH, had a prolonged release time, and prevented the antigenic protein from entering the kidney and spleen, which is essential for an immune response [138].

The mucociliary clearance of drugs is a key issue when delivering pharmaceuticals via the nasal route. Furthermore, due to their low permeability across the nasal epithelium, hydrophilic drugs, proteins, polysaccharides, and nucleic acids pose a challenge. Nasal absorption is essential for the drugs to take effect. The molecular weight, lipophilicity, and charge are physical properties of drugs that influence nasal absorption. A mucociliary clearance occurs for drugs that do not pass the nasal membrane. The development of a mucoadhesive system could be able to aid with this constraint. Chitosan is useful in nasal drug delivery because of its mucoadhesive properties [143]. Nasal absorption occurs via transcellular, paracellular, and trigeminal nerves [144]. Carbamazepine is a drug used to treat epilepsy and must penetrate the blood–brain barrier (BBB). Carboxymethyl-ChNP has improved carbamazepine absorption and brain targeting when administered through the nose. When carbamazepine was administered intranasally loaded in ChNP, the brain-to-plasma exposure ratio was 150% [145]. The gender of a person is a risk factor in Alzheimer’s disease (AD) and 17-estradiol levels in women diagnosed with AD are lower than in healthy ones. Estradiol, a female sex hormone, has been used to prevent and treat AD and it must reach a sufficiently high tissue concentration in the brain to have an effect. Estradiol levels in the cerebral fluid are quite low when taken orally. When estradiol was given intranasally loaded in ChNP, the levels of estradiol in the cerebrospinal fluid were found to be higher than those in plasma. These findings demonstrate that when estradiol is administered via the nasal route loaded in ChNP, it is delivered directly to the brain. Another study indicated that when leuprolide was formulated as thiolated ChNP, the bioavailability of the drug, which is used to treat prostate cancer and hormone-dependent disorders, was improved [146]. When leuprolide was prepared as thiolated ChNP, it enhanced 2–5-fold the drug transport to the porcine nasal mucosa than the leuprolide solution. With thiolated ChNP, drug exposure increased 6.9-fold as determined by the area under the curve (AUC) and the plasma concentration vs. time curve AUC.

Drug delivery to the lungs can have both local and systemic effects. Compared to alternative routes, pulmonary drug delivery has various advantages due to its enormous surface area, high tissue vascularity, and good permeation due to a thin absorption barrier, leading to quick and effective drug delivery [147]. According to Islam et al. [148], pulmonary drug delivery can be achieved using ChNP because the positive charge on the surface of chitosan gives it mucoadhesive characteristics, which is beneficial for pulmonary drug delivery. Chitosan can both improve the uptake of drugs through the lung epithelium and attain antibacterial properties by attaching to lipopolysaccharides and the phosphoryl groups on bacterial cell membranes, which is an advantage for treating bacterial infections in the lungs.

Chitosan was used as the polymer in a rifampicin-loaded nanoparticle dry powder inhalation (DPI) system for the antitubercular drug. This nanoparticle formulation demonstrated sustained drug release for up to 24 h and showed no toxicity to cells or organs. In a mice model, rifampicin displayed better pharmacokinetic parameters, having a higher peak plasma concentration (Cmax), AUC, and longer mean residence time (MRT) [149]. Itraconazole is an antifungal drug with a low solubility when delivered orally so it can be delivered through the lungs to effectively treat pulmonary infections. Also, its properties can be noticeably increased by combining it with lactose, mannitol, and leucine as a spray-dried ChNP. In fact, in this case, a spray-dried formulation can deliver a higher drug concentration to the target, allowing passive targeting and lower systemic toxicity. When itraconazole was formulated as spray-dried microparticles containing itraconazole-loaded ChNP, itraconazole pulmonary deposition was found to be increased (58 ± 2 to 96 ± 1 ng/mL) compared with that of raw itraconazole or an itraconazole microparticle-based DPI (<10 ng/mL) [150].

**Figure 6:**
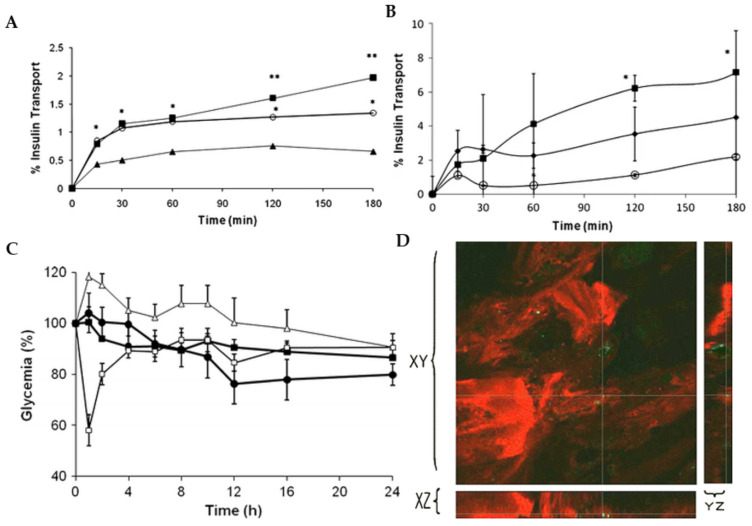
(**A**) Cumulative permeation of insulin through Caco-2 loaded into SLN (circles) and chitosan-coated SLN (squares) comparatively to free insulin (triangles). * *p* < 0.05, SLN and chitosan-coated SLN are statistically different from free insulin. ** *p* < 0.05 chitosan-coated SLN are statistically different from SLN; (**B**) Cumulative permeation of insulin through Caco-2/HT29 co-culture monolayer loaded into SLN (diamonds) and chitosan-coated SLN (squares) comparatively to free insulin (circles). * *p* chitosan-coated SLN are statistically different from free insulin. (**C**) Reduction of plasma glucose concentration upon subcutaneous delivery of insulin 2.5 IU/kg (empty squares), oral delivery of insulin 25 IU/kg (triangles), SLN loading insulin 25 IU/kg (filled squares), and chitosan-coated SLN loading insulin 25 IU/kg (circle) (*n* = 6). (**D**) Green fluorescence of labelled insulin loaded into chitosan-coated SLN in an inner apical intestinal section of a rat.

Another very important capability of chitosan is its ability to coat nanoparticles made by other materials, such as lipids, to improve the delivery properties of the nanocarriers [151]. In a previous work, chitosan-coated solid lipid nanoparticles (SLN) were developed to deliver insulin orally [152]. The nanoparticles had a size of about 450 nm and were positively charged, whereas the uncoated SLN had a negative charge. This modification allows chitosan-coated SLN to better interact with negatively charged cell membranes than uncoated SLN. Therefore, chitosan-coated SLN had higher insulin permeation through a Caco-2 cell monolayer model than uncoated SLN (Figure 6A). Similar behaviour was observed using a Caco-2/HT29 monolayer model, which better resembled the intestinal membrane, showing that the mucoadhesive properties of chitosan enhanced insulin permeation (Figure 6B). After the oral administration of insulin-loaded SLN to diabetic rats, a pronounced hypoglycemic effect was observed after 24 h, which was more evident when chitosan-coated SLN were administered (Figure 6C). Furthermore, fluorescently labelled insulin loaded into chitosan-coated SLN was internalised in enterocytes and localised in the intestinal walls, showing the capacity of the nanocarrier to enhance the intestinal uptake of insulin (Figure 6D).

### 5.2. Cancer Treatment

Chitosan has multidimensional approaches in cancer therapy, such as gene delivery, anticancer drug delivery, and as an adjuvant for vaccines [153]. Due to their unique characteristics, including degradability, biocompatibility, extraordinary cell-membrane permeation, high drug-carrying capacity, pH-dependent therapeutic unloading, multi-functionality, and prolonged residence time in the bloodstream, ChNP have emerged as one of the most promising delivery vehicles for cancer chemotherapy and diagnosis [154]. Exogenous nucleic acid is delivered into tumour cells or the surrounding environment to control the desired gene involved in cancer pathogenesis [155]. Nucleic acid therapies face several challenges during the targeting of the intended tissue, which may restrict their therapeutic effects. Further, cell membrane-based charge repulsion and poor endosomal escape also obstruct nucleic acid treatments. Therefore, nucleic acid delivery systems need to overcome these limitations [156,157].

The major systems employed for gene delivery are viral or non-viral vectors. Although viral vectors are effective transfection agents, their mutagen and carcinogenic qualities limit their application in cancer gene therapy [158]. In recent years, non-viral vectors have been considerably developed as a viable alternative to viral vector nanotechnology [159]. Liposomes, polymer-based carriers, and different nanoparticles are examples of non-viral vectors for gene transfer. Due to their great transfection effectiveness and ease of production, liposomes are one of the most studied gene carriers. However, liposomes have weak encapsulation efficacy, non-specific toxicity, a short shelf life, and limited in vivo stability [160]. Cationic polymers have been widely employed as alternative gene carriers because of their enhanced transfection efficiency, high gene encapsulation, and proton sponge effect [161]. 

However, chitosan is an exception that has no toxicity when used as a gene delivery vehicle. Chitosan has excellent physicochemical qualities that make it suitable for nucleic acid delivery to gene transport. When electrostatic contact with nucleic acids occurs, chitosan rapidly forms complexes, microspheres, or nanoparticles [162]. Because of these promising properties, chitosan is being increasingly researched as a gene delivery vehicle in cancer therapy.

The availability of several free amine groups makes it simple to functionalise chemotherapeutic drugs for conjugation. A succinic anhydride spacer was recently used to conjugate the water-soluble doxorubicin (DOX) to chitosan [163]. The succinic anhydride could react with DOX amine and become carboxylic. DOX carboxylic acid was subsequently conjugated to the free amine groups of chitosan using carbodiimide chemistry. Then, chitosan-DOX was self-assembled into nanoparticles in an aqueous solution while stirring at room temperature. In addition, adding additional DOX lowered the conjugation efficiency of chitosan. Trastuzumab, a monoclonal antibody that targets Her2+ (human epidermal growth factor receptor 2+), was also attached to chitosan-DOX NP by thiolation of lysine residues by interacting with primary amines and the subsequent attaching of the thiols to chitosan. Compared to chitosan-DOX and free drugs, trastuzumab coupled with chitosan-DOX NP exhibited target specificity towards Her2+ cancer cells and, subsequently, increased absorption occurred. Furthermore, trastuzumab-conjugated chitosan-DOX NP distinguished between Her2+ and Her2 cells with high efficiency, showing their potential for use in active targeted drug delivery.

Chitosan derivatives with suitable properties that can hold hydrophobic drugs have been synthesised to deliver poorly water-soluble drugs. When encapsulated in glyceryl monooleate-chitosan core–shell nanoparticles produced using an emulsification–evaporation process, paclitaxel, a hydrophobic drug, showed increased antitumor activity [164]. In MDA-MB-231 human breast cancer cells, this core–shell nanosystem resulted in a 1000-fold reduction in paclitaxel IC_50_ (half-maximal inhibitory concentration). This significant decrease in the IC_50_ value would lessen paclitaxel cytotoxicity in normal cells. In another study, Kim et al. [165] developed an amphiphilic chitosan derivative for paclitaxel delivery. To produce nanoparticles, they blended glycol chitosan with 5β-cholanic acid (glycol chitosan hydrophobically modified with 5β-cholanic acid or human chorionic gonadotrophin (HCG) nanoparticles, human chorionic gonadotrophin). The drug loading achieved for paclitaxel in the HCG nanoparticles was 80% compared to the traditional Cremophor EL formulation used for paclitaxel delivery, and the cytotoxicity of HCG nanoparticles was insignificant.

Tumour-specific ligands have been coupled to ChNP for targeted drug delivery [166]. ChNP are used to target surface receptors that are increased in cancer cells for receptor-targeted chemotherapy. Nanoparticles with specific targeting ligands generate receptor-mediated endocytosis nanoparticles when they contact cell surface receptors. Because the levels of expression of these receptors differ based on the kind of cancer, knowing the receptor levels and cell types is necessary for designing customised drug carrier systems. Moreover, ChNP with tumour-specific ligands through pH-cleavable bonds, break down the assembly at the endo-lysosome acidic pH and the drug is released into the cytoplasm [167].

Adjuvants are used in vaccines to boost the immune response. However, because of the potential for side effects, scientists are looking for harmless and effective adjuvants for vaccine fabrication, particularly in cancer therapy. Polysaccharides derived from plants, animals, and fungi have been considered cancer vaccine adjuvants [168]. Because of its safety, cationic nature, biocompatibility, and capacity to be used as an antigen carrier, chitosan can be an excellent adjuvant for vaccines [169]. Chitosan’s immunostimulatory properties have been known for more than twenty years. However, its potential as a non-toxic and safe adjuvant in cancer vaccine development has only recently been discovered and chitosan’s adjuvant qualities in cancer and infectious disease vaccines have only recently been examined [169]. Bio-adhesive characteristics of chitosan facilitate cell absorption, resulting in powerful systemic and mucosal immune responses. Chitosan has the unique ability to stimulate cell-mediated and humoral immune responses [170]. Chitosan’s immunological activity is comparable to incomplete Freund’s adjuvant and is better than aluminium hydroxide (Imject™Alum), a common immunoadjuvant [171]. Chitosan helps to keep the longer administration time of peptide antigen at the site, allowing more efficient immune activity. Even after 7 days, Zaharoff et al. found that over 60% of the antigen is engaged in the subcutaneous injection site [172]. This method can reduce the number of vaccination doses required for an improved immune response. One of the studies found that ChNP boosted OVA-induced Th1 and Th2 immune responses in mice [173]. ChNP improved Th1 (IL-2 and IFN-γ) and Th2 (IL-10) cytokine levels, but they also improved natural killer cells’ activity. As a result, chitosan could be a safe and efficient immunoadjuvant for cancer vaccines by increasing cellular and humoral immune responses.

### 5.3. Tissue Engineering

Due to their unique properties, biologically active natural materials have recently received attention as viable materials for tissue engineering. Because of their physical and chemical similarities, they can mimic the structure of human tissue. Natural bioactive materials have been more popular in recent decades since natural polymers are less hazardous and more biocompatible than most synthetic polymers. Repairing wounded tissue is still a big challenge so better and more durable biomaterials must be developed for tissue engineering. Several natural and synthetic materials have been employed for tissue engineering applications including chitosan, collagen, alginate, hydroxyapatite, etc. [174]. However, these constituents may not fulfil the demands of tissue engineering due to limitations such as uncontrolled breakdown, infection risk, poor mechanical qualities, difficulty in the accumulation of degraded biological products, and acidic environments. Hybrid biocomposites with exceptional characteristics have been developed to overcome these obstacles. Two or more biopolymers can be placed together to overcome the limitations of single-component composites. Chitosan has been extensively explored as a possible bioactive material and it has been recognised as an excellent product for tissue engineering, especially for repairing wounded and deceased tissues due to the presence of amino, hydroxyl, and carboxyl functional groups, which aid in the easy formation of composite systems with other natural and synthetic materials. This enhances the biological and mechanical properties of chitosan that can be exploited for the treatment of acute and chronic wounds and other skin tissue engineering applications [175].

Additionally, chitosan has very potent hemostatic properties that are reliant on the molecular weight and degree of deacetylation of the substance rather than the host coagulation pathway [176] or deacetylation [177,178]. In addition, it has a variety of other effects on all phases of healing, including causing neutrophils to migrate [179] and neutrophil-like HL60 cells to release IL-8, a strong neutrophil chemokine, in response to chitosan in direct proportion to the degree of N-acetylation [180]. Chitosan also has an immunomodulatory effect since it causes macrophages to produce inflammasomes when exposed to micro- and nanosized chitosan particles [181,182]. The use of macro-sized chitosan scaffolds is sensible when there is excessive inflammation; however, macro-sized chitosan inhibits IL-1 production and activation of inflammasomes in mouse and human macrophages in vitro [183]. Additionally, chitosan impacts growth factor expression by altering TGF-1 expression in the later phases by adhering to anionic growth factors [184] and boosting it in the early post-injury phase [38]. It promotes cutaneous fibroblast growth, enabling the development of fibrous tissue and re-epithelialisation [185]. Without the need for any cross-linking agents, a complex chitosan-cordycepin hydrogel was recently created using a freeze-drying technique in which negatively charged cordycepin was attached to positively charged chitosan chains [160]. Chlorhexidine was also stuffed into a textile polyethylene terephthalate by coating with chitosan, and post-thermal treatment enhanced its mechanical stability by lengthening the time it took for chlorhexidine to release into the environment by up to 7 weeks [186]. Chitosan has also been employed in asymmetric membranes, either by itself or in combination with other natural polymers, typically as an underlayer that comes into touch with the damaged skin [187]. Another method for creating biomaterials is the addition of nanoparticles to hydrogels [188]. Shah et al. created a moxifloxacin-loaded, triple-component nanocomposite film that contains chitosan, silver, and sericin. In addition to having strong antibacterial action against clinical isolates of methicillin-resistant *Staphylococcus aureus* (MRSA), the resulting films also promoted wound healing in rats, much like commercial wound dressings [189]. Most chitosan composite films, including collagen, have inherent abilities to promote healing, for instance, a human keratin-chitosan membrane created using the UV cross-linking process exhibited potential as a wound dressing [190]. The effective antibacterial action and cytocompatibility mean the chitosan-chondroitin sulphate-based polyelectrolyte complex is appropriate for use in wound healing [191]. Additionally, growth factors and cytokines could be added to positively charged biomaterials containing chitosan to enhance their effectiveness in the wound healing process. In a recent work, granulocyte-macrophage colony-stimulating factor (GM-CSF) was loaded onto ChNP made using ionotropic gelation with tripolyphosphate [192] as a component of a nanocrystalline cellulose-hyaluronic acid composite made using the freeze-drying method [193]. In vivo tests have shown that composite material embedded with GM-CSF in ChNP promoted the healing process more effectively than the composite by itself, with a controlled delivery over 48 h and the loading efficiency was as high as 97.4 ± 1.68% [50]. Accelerated wound closure was also demonstrated by polycaprolactone nanofibers loaded with ChNP containing GM-CSF [194]. Chitosan hydrogels made from Ser-Ile-Lys-Val-Ala-Val-chitosan macromers boosted collagen appearance, angiogenesis, and TGF-1 expression while decreasing TNF-, IL-1, and IL-6 mRNA expression [176,195], all of which contributed to wound closure in vivo in mouse skin. To boost affinity for the growth factors, chitosan can undergo additional modifications, for instance, creating heparin-like polysaccharide (2-N, 6-O-sulfated chitosan), which has a higher affinity for the vascular endothelial growth factor than heparin because of its higher sulfonation degree [196,197].

Neurodegenerative diseases and brain and spinal cord injuries are central nervous system (CNS) problems caused by damage to axons in the brain and spinal cord [177]. The regeneration of injured tissue in the CNS is difficult due to its limited ability to repair [178]. Tissue engineering is a combinatorial approach for healing injured tissues that has recently been applied to repair or replace missing neural tissues. The ChNP in tissue engineering serve as a platform for cell growth, resulting in the formation of a specific tissue with specified functionalities. As an inductive milieu for brain regeneration, many natural and synthetic biomaterials have been developed [179]. Conductive polymers are one of the most attractive options because they produce electrical impulses such as those found in natural nerve tissues. Electrically conductive-based scaffolds can be employed to reconstruct nerve, muscle, and heart tissue [180]. 

It is essential to develop biocompatible and biodegradable biomaterials with appropriate mechanical properties and interconnected pores [174,181] that support cell differentiation [182]. However, it is difficult to make a biomaterial with such qualities using a single polymer. Thus, hybrid materials need to be developed, which could be suitable for bone and cartilage regeneration [183,184]. ChNP for hard tissue regeneration have weak mechanical stability when wet, needing additional adjustments [185,198]. Because chitosan favours calcium/phosphate ion build-up and boosts the biomineralisation potential of polyethylene glycol diacrylate/chitosan-based hydrogels, this characteristic of the polymer has been used to improve the biomineralisation of hybrid materials [186]. 

Because of its biological resemblance to the inorganic component of bone, hydroxyapatite is commonly used to improve the mechanical qualities of chitosan [187]. In addition to hydroxyapatite, other hybrids with equivalent mechanical properties have been created such as nano-calcium zirconate/chitosan and strontium-modified chitosan/montmorillonite hybrids [188,189]. A chitosan/chondroitin/nano-bioglass-based polyelectrolyte hybrid material with improved bioactivity, such as apatite build-up and increased type-1 collagen expression by MG63 osteoblast-like cells in vitro, as well as scaffold osteointegration in vivo, has been developed [186]. Chitosan has active biomineralisation properties that could be improved by incorporating additional polymers such as fucoidan [191] and bioglass [192].

In modern orthopaedics, regenerating cartilage that has been injured through accident, disease (osteoarthritis), or ageing is a critical undertaking. Microfracture, mosaicplasty, autologous chondrocyte, and biomaterial implantation are some methods used to regenerate cartilage [193,194]. The lack of blood arteries in cartilage tissue is a significant constraint; thus, the main goal of tissue engineering is to develop a biomaterial capable of stimulating cartilage regeneration under avascular conditions [193,195].

### 5.4. Antibacterial Activity

The antibacterial activity of ChNP is due to their interactions with either the bacterial cell wall or the cell membrane. Several hypotheses have been proposed to explain this framework. The electrostatic communication between the amino groups of glucosamine (positively charged) and the cell membranes of bacteria (negatively charged) is perhaps the most commonly accepted ChNP model of antimicrobial action [199]. This interaction causes widespread changes to the cell surface, resulting in a change in membrane permeability, which, in turn, causes osmotic imbalance and efflux of intracellular substances, resulting in cell death [196,200]. Furthermore, ChNP have the potential to adjust the electron transport chain of bacteria [197]. One more likely mechanism is chitosan’s ability to chelate metal ions, which stimulates toxin production while preventing bacterial viability [201]. In acidic conditions, chitosan sustains significantly larger chelating activity for various metal ions (including Fe^2+^, Mg^2+^, Ni^2+^, Co^2+^, Cu^2+^, and Zn^2+^). The normalisation of the cell wall particles of bacteria varies depending on the metal ions. As a result, chelating of these metal ions by chitosan has often been thought of as a potent antimicrobial pathway, which is more effective at high pH levels where positive ions are captured by chitosan since the NH_2_ groups are unprotonated and the electron pair on the amine nitrogen is available for donation to the metal ions. Chitosan molecules undeniably surround the metal complex and obstruct vital nutrient flow, resulting in cell death. As a result, the appropriate deployment of ChNP is dependent on a variety of variables that can be changed [202].

Chitosan has diverse applications in a range of industries, from biomedical to cosmetic and food industries. This has resulted in the development of a wide range of chitosan-containing formulations. [203]. Hipalaswins et al. demonstrated the antibacterial activity of synthesised ChNP against clinically pathogenic bacterial strains such as *Proteus mirabilis, Pseudomonas fluorescens*, *Staphylococcus aureus*, *Klebsiella pneumoniae*, *Escherichia coli*, and *Enterobacter aerogenes* [204]. The latter was found to be the most susceptible, followed by *E. coli*, *K. pneumoniae*, *P. fluorescens*, and *P. mirabilis*. The ChNP were found to be less toxic toward *S. aureus*. Similarly, ChNP incorporated with lime essential oil exhibited antibacterial activity against four pathogens (*S. aureus*, *Listeria monocytogenes*, *Shigella dysenteriae*, and *E. coli*). *S. dysenteriae* was highly sensitive to ChNP and showed the highest susceptibility [205]. Curcumin-loaded ChNP can be used both in drug delivery and as an approach to accurately stimulate antibacterial mechanisms as they inhibited the growth of *P. aeruginosa* and *S. aureus* infections in mice [206]. Pilon et al. demonstrated that ChNP coated with citric acid hindered the growth of mesophilic microorganisms more effectively than conventional coating [207]. The ChNP (110 nm) coating was proven to be more efficient at impairing microbial growth. This study validated the use of ChNP as edible films for bacterial growth regulation in fresh fruit and vegetables. Therefore, the smaller the ChNP, the higher the surface interaction and motion, and the higher the antimicrobial property against disease-causing microorganisms.

## 6. Conclusions and Future Perspectives

According to recent developments, chitosan is one of the most explored bio-based polymers. The FDA has granted this substance GRAS (Generally Recognised as Safe) status as a food ingredient, indicating that it is safe. Due to its biocompatibility and biodegradability, chitosan has a wide range of applications, with special emphasis on biomedical engineering and drug delivery systems. Chitosan is also used in farming, the food industry, water treatment, pollution control, photography, papermaking, and others. Furthermore, the positive surface charge and mucoadhesive properties of nanoparticles made of chitosan and chitosan derivatives enable them to attach to mucous membranes and release the loaded drug over time. These ChNP have several uses in non-parenteral drug administration for the treatment of eye infections, cancer, gastrointestinal illnesses, respiratory diseases, and cancer due to their physical features and lack of toxicity. Importantly, chemically modified chitosan can help to improve in vivo transfection efficacy, whereas naturally occurring ChNP with nucleic acid have a limited capacity for buffering and endurance. Furthermore, a wide range of other forms, such as antiviral drugs, proteins, peptides, nucleic acids, and even fully inactivated viruses, can be included in the chitosan matrix. They can enhance cellular absorption and increase drug or gene delivery to the site of viral infection. In addition, ChNP have attracted attention in the field of nanomedicine for the creation of novel therapeutic drug release systems due to their enhancement of the bioavailability of drugs and their specificity, sensitivity, and lower toxicity. Despite the potential advantages of chitosan in drug delivery or tissue engineering systems, its poor long-term reliability is a significant barrier to scaling-up chitosan pharmaceutical formulations. Aside from the type of polyanion used, a variety of technological factors, such as pH changes, charge density, or polymer concentration, could ascertain or attenuate the biomedical applications or could raise safety concerns for human use. Therefore, more studies, especially in the nasal and pulmonary administration of drugs as an innovative approach for bypassing chitosan’s constraints for a wider variety of pharmaceutical drugs and even macromolecules, are urgently needed. Furthermore, the wide range of ChNP has shown therapeutic potential in a variety of neurological diseases. This chitosan and its derivatives as nano-biodegradable carriers could contribute significantly to brain drug delivery due to their fine biological properties, extensibility, and efficacious uptake by intranasal mucosal cells to tumour cells. Finally, industrial advancements in anticancer drugs, gene delivery, catalysis, sensor applications, wrapping materials and packaging, cosmetotextiles, and bioimaging are also underway.

## Figures and Tables

**Table 1:** Findings of chitosan-based nanoparticles used in biomedical applications.

Chitosan Nanoparticles	Biomedical Application	Findings	References
Ch-Au particles	Biomedical sensors	Immobilisation of enzymes	[119]
Ch–montmorillonite nanocomposites	Biomedical sensors	Used for anionic detection in aqueous samples	[120]
Ch-RNAi complexes	Gene therapy	Transfection of CHO-K1, HEK293, H1299, HepG2 cells	[121]
Ch-grafted polyethylene glycol methacrylate	Ophthalmic diseases	No cytotoxicity, hemocompatible	[122]
Graphene/AuNP/Ch electrode	Glucose biosensor	High electrocatalytic activity toward hydrogen peroxide and oxygen	[123]
Insulin-loaded lecithin/ChNP	Drug delivery system	Increased bioavailability, release, and enhanced therapeutic properties	[124]
Chitin nanofiber composite	Therapeutic enzyme immobilisation	Separation of immobilised chymotrypsin is easy and recycled	[125]
Modified glycol ChNP-encapsulated camptothecin	Cancer therapy	Efficient drug delivery system	[126]
Palladium NP chitosan oligosaccharide with RGD peptide	Breast cancer therapy by enhancing photothermal effects	Enhanced imaging and tumour therapy	[127]
Saquinavir-loaded ChNP	Anti-HIV system	Strains of HIV—NL4-3 and indie-C1 responded to the delivery system	[128]
Sodium alginate with Ch and olive oil-coated beads	*Helicobacter pylori* infections	Controlled release of active clarithromycin	[129]
Timolol maleate-galactosylated ChNP	Ocular delivery of timolol maleate	Enhanced penetration and retention	[125]
Zinc-ChNP	Acute lymphoblastic leukaemia	Induced apoptosis in human acute T-lymphocyte leukaemia	[130]

NP stands for nanoparticles.

## Data Availability

Not applicable.

## Abbreviations

AD—Alzheimer’s disease; AUC—Area under curve; BBB—Blood-brain barrier; BSA-Bovine serum albumin; CNS—Central nervous system; CCD—Charge-coupled device; ChNP—Chitosan nanoparticles; DOX—doxorubicin; DPI–Dry powder inhalation; ECM—Extracellular matrix; FDA–Food and Drug Administration; GM-CSF-Granulocyte-macrophage colony-stimulating factor; HGC NP—Hydrophobically modified glycol chitosan nanoparticles; IL—Interleukin; NIRF—Near-infrared fluorescence; PANI—polyphenylene; PBS—Phosphate-buffered saline; PLGA—Poly(lactic-co-glycolic acid); PPy-Alg—Polypyrrole-alginate; PPy—Polypyrrole; PTh—Polythiophene, SEM—Scanning electron microscopy; SLN—Solid lipid nanoparticles; TE—Tissue engineering; TEM—Transmission electron microscopy; XPS—X-ray photoelectron spectroscopy.
